# Use of Personalized Ultra-Fractionated Stereotactic Adaptive Radiotherapy for Oligometastatic Lung Adenocarcinoma: Leveraging CT-Guided Online Adaptive Radiotherapy

**DOI:** 10.7759/cureus.66877

**Published:** 2024-08-14

**Authors:** Nicholas Eustace, Colton Ladbury, Yufei Liu, Arya Amini, Sagus Sampath, Tyler Watkins, Kevin Tsai, Borna Maraghechi, Chunhui Han, Chengyu Shi, An Liu, Terence Williams, Percy Lee

**Affiliations:** 1 Radiation Oncology, City of Hope National Medical Center, Duarte, USA; 2 Radiation Oncology, City of Hope Orange County Lennar Foundation Cancer Center, Irvine, USA

**Keywords:** radiotherapy, pulsar, online adaptive, ct guided, sbrt, oligometastatic, nslcl

## Abstract

Management of oligometastatic non-small cell lung cancer (OM-NSCLC) has changed considerably in recent years, as these patients were found to have better survival with systemic therapy followed by consolidative radiation. Stereotactic body radiotherapy (SBRT), characterized by high doses of radiation delivered in a limited number of fractions, has been shown to have improved local control compared to conventionally fractionated radiation in early-stage lung cancer, but its use in large tumors, ultra-central tumors, or mediastinal nodal regions is limited due to concerns of toxicity to nearby serial mediastinal structures. Recent improvements in image guidance and fast replanning allow adaptive radiotherapy to be used to personalize treatment to the patient's daily anatomy and ensure accurate dose delivery to the tumor while minimizing dose and toxicity to normal. Adaptive SBRT can expand its use into ultra-central tumors that otherwise may not be amenable to SBRT or enable alternative fractionation schedules such as personalized ultra-fractionated stereotactic adaptive radiotherapy (PULSAR) with one-month intervals between fractions. In this case, we report a patient initially presenting with bulky OM-NSCLC of the left lung and mediastinum with an isolated left femur metastasis who was referred for consolidative radiotherapy after systemic therapy. We demonstrate how CT-guided online adaptive radiotherapy to the lung and mediastinum can be used despite the long time interval between treatments. In addition, adaptive plans lead to a substantial decrease in the heart dose, with moderate decreases in other organs compared to non-adaptive plans. This case demonstrates the feasibility of using adaptive radiotherapy for PULSAR of ultra-central OM-NSCLC.

## Introduction

Oligometastatic non-small cell lung cancer (OM-NSCLC) is defined by the European consensus statement as NSCLC that has spread to a maximum of five other sites in up to three organs and is estimated to occur in 20%-50% of new diagnoses [1]. The management of OM-NSCLC has changed considerably in recent years, as these patients were found to experience improved survival rates when treated with systemic therapy followed by consolidative radiation [2]. Even with the development of effective targeted agents, local treatment with radiation has continued to show survival benefits [3,4]. Before modern improvements in image guidance, conventionally fractionated radiation was delivered with multiple daily fractions of small doses to a large area, relying upon differences in DNA damage repair between tumor and normal tissues to provide a therapeutic window [5].

Stereotactic body radiotherapy (SBRT), characterized by high doses of radiation delivered in a limited number of fractions [6,7], has been shown to have improved local control compared to conventionally fractionated radiation over six to seven weeks in early-stage lung cancer [8,9], but its use in large or ultra-central tumors has been limited due to concerns of toxicity to nearby structures. Additional improvements in fast replanning have allowed for adaptive radiotherapy to be utilized that personalizes treatment to the patient's daily anatomy, allowing for accurate dose delivery to the tumor while minimizing dose and toxicity to normal structures [10,11]. This ability to adapt treatment to the current anatomy can allow SBRT to be utilized more safely near at-risk normal structures. A new method of improving radiotherapy outcomes under investigation utilizes extended periods between the high-dose fractions of SBRT in a technique called personalized ultra-fractionated stereotactic adaptive radiotherapy (PULSAR) [12]. The time between the fractions of PULSAR can vary from one week to one month, and this extended time delay between treatments provides more time for normal tissues to heal and for tumor(s) to shrink away from critical normal tissue. This has the potential to decrease the toxicities associated with high dose-per-fraction treatments and enable its use in patients otherwise unable to receive SBRT due to tumor size or location.

Furthermore, PULSAR may improve responses to radiotherapy alone or in combination with immunotherapies by allowing for tumor microenvironmental changes or immune activation to occur in between fractions, and alternative fractionation schedules are an active area of investigation [13,14]. However, the anatomical changes that can occur during the long intervals between fractions require replanning with each treatment, which can limit its adoption. In this case, we present the use of CT-guided online adaptive radiotherapy to deliver PULSAR for consolidative treatment of OM-NSCLC in an ultra-central location that otherwise would prevent its use.

## Case presentation

**Figure 1:**
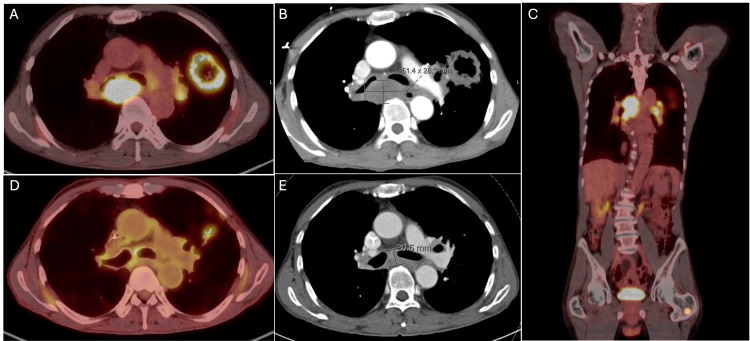
Oligo-metastatic adenocarcinoma of the lung The pre-chemotherapy imaging shows the fluorodeoxyglucose (FDG) avid mass in the left lung and mediastinum seen on (A) axial PET-CT, (B) axial CT, and (C) sagittal PET-CT, also showing a left femur metastasis. Subsequent post-chemotherapy imaging revealed a decrease in the size and FDG avidity of mediastinal and left lung disease on (D) axial PET-CT and (E) axial CT. PET-CT: positron emission tomography/computed tomography.

A 73-year-old man with a 20-pack-year smoking history was diagnosed with OM-NSCLC T3N3M1b after routine imaging revealed a lesion in the left upper lung, and a subsequent fluorodeoxyglucose (FDG) positron emission tomography (PET)/computed tomography (CT) revealed a necrotic left upper lung lesion measuring 6.6 cm in size. The scan also showed the presence of numerous aggregated left hilar, mediastinal, and subcarinal adenopathies with isolated metastasis to the left proximal femur. The MRI brain revealed no metastasis. A biopsy of these lesions confirmed poorly differentiated invasive adenocarcinoma of lung origin, and the next-generation sequencing showed a high mutational burden with mutations in *PIK3CA* (E545K), *TP53*, and *MET* (L1195V), as well as *PDL1* expression of 90%. He received four cycles of carboplatin, pemetrexed, and pembrolizumab, with a good response seen on CT showing a decrease in the size of the cavitary left upper lobe mass and no new metastases (Figure 1).

**Figure 2:**
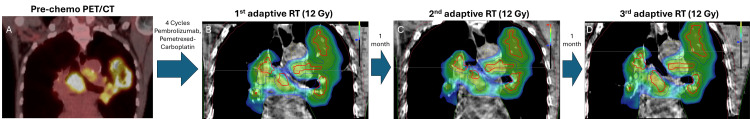
Timeline of PULSAR with one-month intervals between radiotherapy fractions (A) Coronal image of pre-chemo positron emission tomography (PET) and coronal images of computed tomography (CT) of the (B) first, (C) second, and (D) third fractions demonstrating the timing of treatments (one-month interval) and dose per fraction (12 Gy). Gross tumor volume (red); planning target volume (blue); heart (green).

Given the patient's good response to systemic therapy and plan for concurrent immunotherapy, we favored a high dose-per-fraction ablative therapy such as SBRT over hypofractionated RT to maximize potential immunotherapy responses to his OM-NSCLC. However, since SBRT would be prohibitive due to the size and central location of his mediastinal and lung disease due to potential grade V toxicity, we proceeded with PULSAR to a planned dose of 36 Gy delivered in three fractions one-month fractions with concurrent pembrolizumab. Adaptation was necessary for PULSAR to account for anatomical changes between the one-month fractions to treat the lung and mediastinal lesions while minimizing the dose delivered to the esophagus and heart (Figure 2).

Non-adaptive treatment was used for the metastatic left femoral lesion given minimal motion of the lesion and no nearby dose-limiting structures. The three-fraction PULSAR treatments to both the lung and femur were completed without interruption. Pembrolizumab was continued throughout the first and second PULSAR treatments but held at the time of the third PULSAR treatment since he was noted to have Radiation Therapy Oncology Group (RTOG) grade 1 fatigue and cough, but no dyspnea or esophagitis. At the one-month follow-up, the patient was given a four-week 40 mg prednisone taper for RTOG grade 2 pneumonitis with subsequent improvement in his symptoms. It was unclear if the pneumonitis was due to immunotherapy, PULSAR radiotherapy, or the combination due to the timing. At the most recent follow-up six months after completion of treatment, the patient showed no signs of disease progression.

Simulation, treatment planning, and delivery

**Figure 3:**
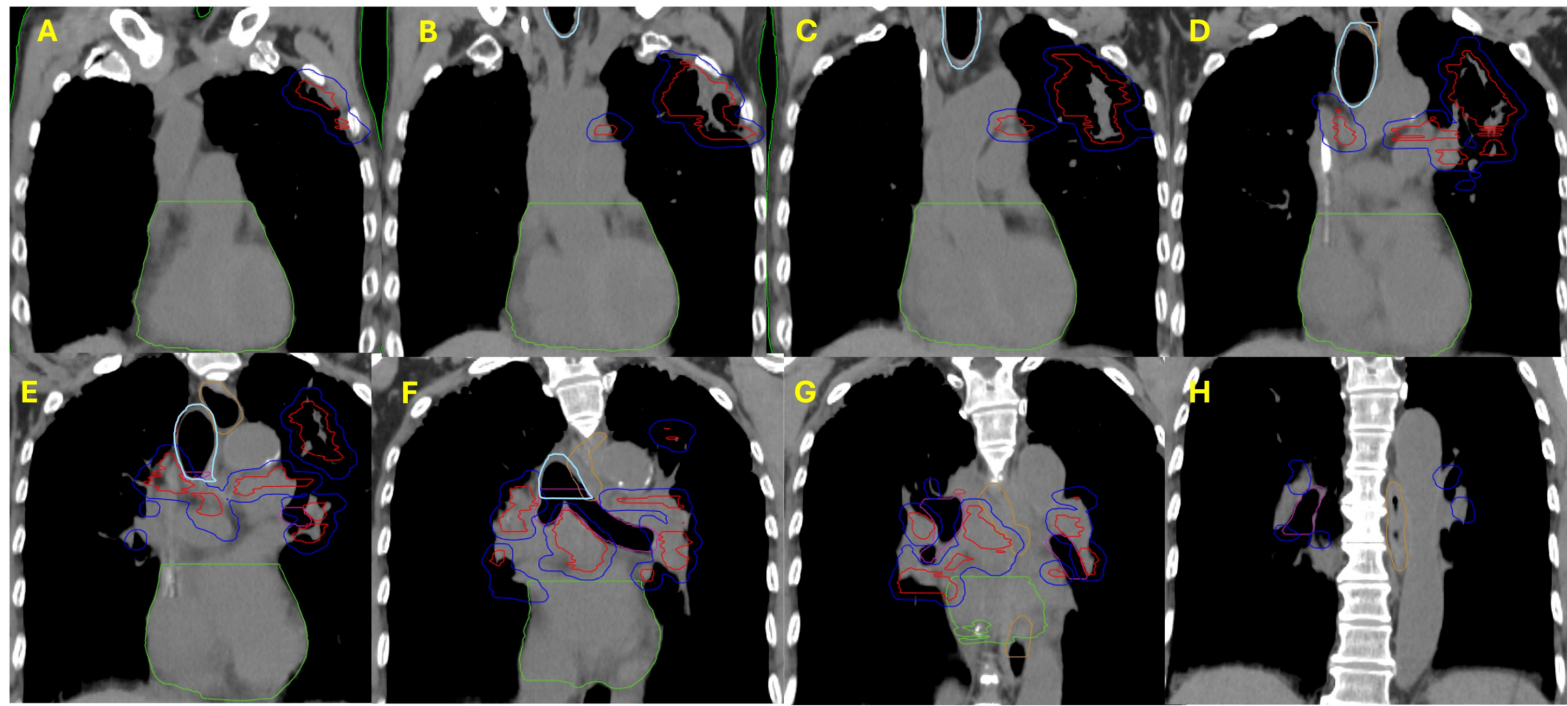
Planning CT contours of the left lung and mediastinal non-small cell lung cancer (NSCLC) Coronal planning CT slices with contours display gross tumor volume (red), planning target volume (blue), heart (green), trachea and large bronchus (light blue), esophagus (orange), and bronchus/small airway (pink). Slices are shown every 1 cm and go from the anterior to the posterior from the (A) anteriormost slice, showing the anterior extent of the disease, which extends towards the second and third ribs in the left lung. Additional posterior slices (B, C) reveal bulky left lung and left mediastinal disease, and slices (D-G) demonstrate the disease extending to the right mediastinum. The most posterior CT slice (H) depicts the posterior extent of the planning target volume, with no additional gross tumor volume.

The patient was immobilized using a Vac-Lok mold (CIVCO Radiotherapy, Orange City, Iowa) with arms overhead and received a four-dimensional CT (4DCT) for treatment planning. The treatment planning targets and organs at risk were contoured by an experienced radiation oncologist on the CT average, and a gross tumor volume (GTV) was defined using PET and CT imaging (total volume of 103 cm^3^). No internal GTV (iGTV) or internal target volume (ITV) was used due to the treatment volume and limited motion on breathing assessment. No clinical target volume (CTV) was defined per our SBRT protocol. A uniform 5-mm planning target volume (PTV) expansion (total volume of 309 cm^3^) was made (Figure 3).

In addition to the GTV and PTV, planning optimization volumes of PTV-GTV, PTV_OPT (PTV - (bronchus small airway + 0.3 cm) - (heart/pericardium + 0.2 cm) - trachea large bronchus), and GTV_OPT (PTV-OPT - 0.3 cm) (volumes of 205 cm^3^, 273 cm^3^, and 145 cm^3^, respectively) were generated to deprioritize target coverage that overlaps with organs at risk (OAR) given the size and location of the tumor and ensure adequate dose to planned targets. A total of 13 OARs were defined including bronchus small airway, esophagus, great vessels, heart/pericardium, skin, spinal cord, trachea large bronchus, lung, ribs, brachial plexus, body, left lung, and right lung (Table 1).

**Table 1:** List of planning target volumes and organs at risk GTV_OPT: gross tumor volume optimized; PTV: planning target volume; CT: computed tomography; PET: positron emission tomography.

Target Volumes and Organs at Risk	Definition	Derivation
GTV	Gross tumor volume	Visible tumor on CT or PET
PTV	Planning target volume	GTV + 0.5 cm
PTV-GTV	Planning target volume minus gross tumor volume	PTV - GTV
GTV_OPT	Optimized gross tumor volume	PTV_OPT - 0.3 cm
PTV_OPT	Optimized planning target volume	PTV - (bronchus small airway + 0.3 cm) - (heart/pericardium + 0.2 cm)
Body	-	-
Brachial plexus	-	-
Bronchus/small airway	-	-
Esophagus	-	-
Great vessels	-	-
Heart/pericardium	-	-
Left lung	-	-
Lung	-	-
Ribs	-	-
Right lung	-	-
Skin	-	-
Spinal cord	-	-
Trachea/large bronchus	-	-

**Figure 4:**
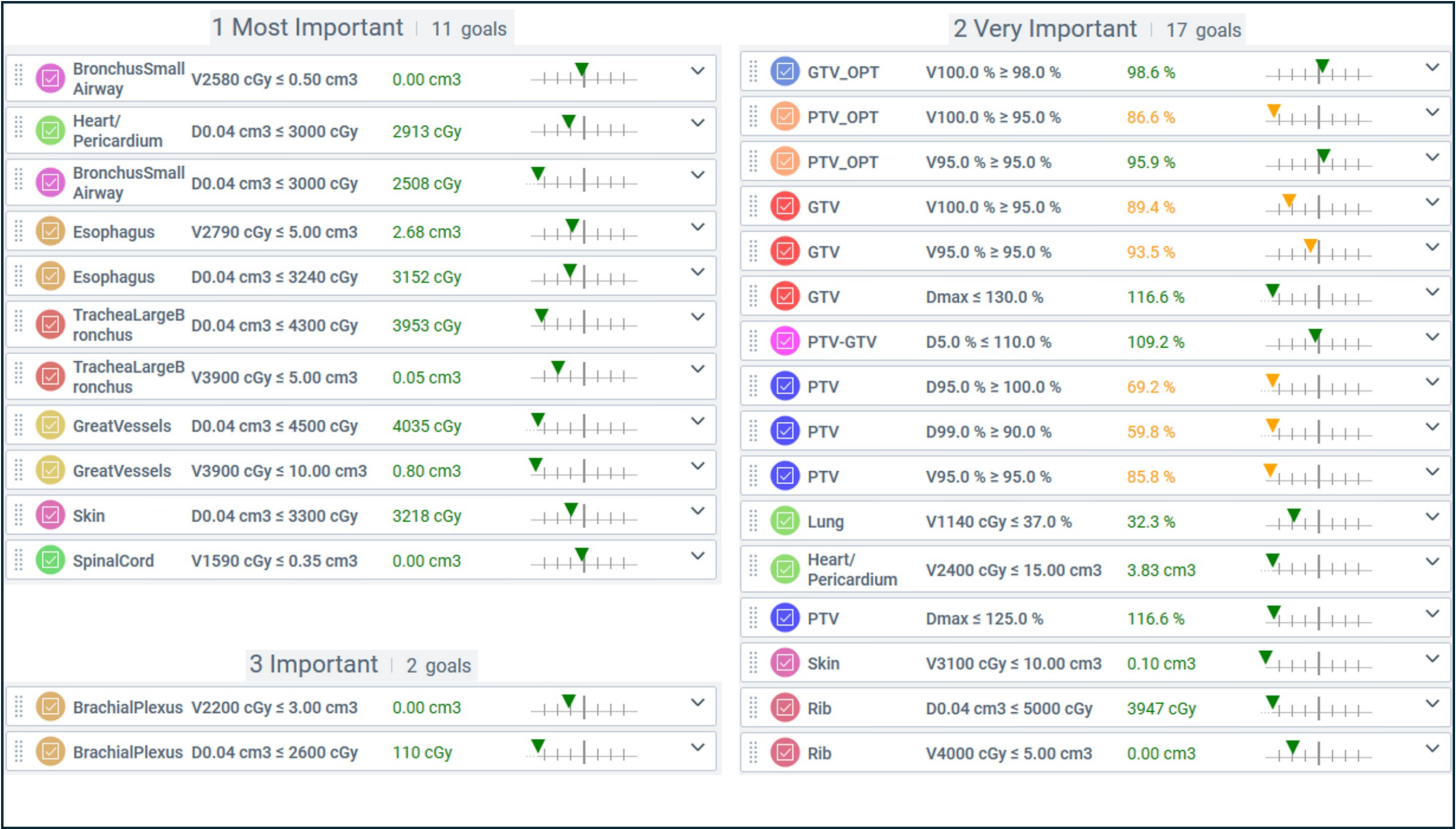
ETHOS planning objectives and respective reference plan values in order of importance GTV: gross tumor volume; PTV: planning target volume; PTV_OPT: planning target volume optimized (PTV – (Bronchus Small Airway +0.3 cm) – (Heart/Pericardium + 0.2 cm) – Trachea Large Bronchus); GTV_OPT: gross tumor volume optimized (PTV_OPT – 0.3 cm); Dmax: maximum dose as percent (%) of the prescription dose; Vxx Gy: volume (cm^3^ or %) receiving xx centigray (cGy); D0.04 cm^3^: point volume (0.04 cm^3^ ) receiving xx centigray (cGy).

We prioritized our planning goals in Ethos^TM ^into the categories Most Important, Very Important, and Important**.** No goals were defined for the body, left lung, or right lung, and no goals were categorized as Less Important. Both the reference and adaptive plans were generated prioritizing OAR constraints over target coverage. Given the size and central location of the tumor, the optimization GTV (GTV_OPT) was prioritized with V100% Rx >98% (Figure 4).

At the time of CT-guided online adaptive treatment, structure contours were adjusted by an experienced radiation oncologist based on changes in anatomy seen on thorax protocol cone-beam CT. The change in volume of the GTV ranged from -1% to +8% over the three treatment sessions compared to the planning scan, and PTV ranged from -3% to +3%. The changes in volume to other planning volumes and select OARs are shown in Table 2.

**Table 2:** Contour volumes and the percent change in the adapted volumes compared to the reference planning volumes GTV: gross tumor volume; PTV: planning target volume; PTV_OPT: planning target volume optimized (PTV – (Bronchus Small Airway +0.3 cm) – (Heart/Pericardium + 0.2 cm) – Trachea Large Bronchus); GTV_OPT: gross tumor volume optimized (PTV_OPT – 0.3 cm).

Target	Planned Volume (cm^3^)	Session 1		Session 2		Session 3	
		Structure volume (cm^3^)	% Change	Structure volume (cm^3^)	% Change	Structure volume (cm^3^)	% Change
GTV	103.1	101.92	-1%	102.57	-1%	110.87	8%
PTV	308.6	300.74	-3%	305.17	-1%	319.25	3%
PTV-GTV	205.5	198.75	-3%	202.55	-1%	208.36	1%
GTV_OPT	144.99	143.61	-1%	149.07	3%	155.21	7%
PTV_OPT	273.12	267.03	-2%	274.21	0%	288.2	6%
Heart	580.93	681.19	17%	582.95	0%	589.06	1%
Trachea large bronchus	59.76	58.03	-3%	57.77	-3%	58.8	-2%
Bronchus small airway	31.56	32.26	2%	30.67	-3%	30.56	-3%

The adaptive plan was used for treatment if a violation of at least one OAR hard constraint was solved compared to the reference plan. All these fractions required adaptive treatment as there was a violation in the heart/pericardium and bronchus/small airway while preserving PTV coverage. In brief, the cumulative heart constraint of V2400 cGy ≤ 15 cm^3^ was exceeded in the first fraction by the non-adapted scheduled plan (22.12 cm^3^) compared with the adaptive plan (4.29 cm^3^). Furthermore, the cumulative point dose constraint of D0.04 cm^3^ ≤ 3000 cGy and the single fraction limit of 1000 cGy were exceeded in each of the three non-adapted fractions (1351 cGy, 1177 cGy, 1195 cGy, respectively) as well as the cumulative dose (2997 cGy), whereas the adapted fractions were able to adhere to these limits (999 cGy in all three individual fractions and 2997 cGy cumulative dose). The bronchus/small airway constraint of V2580 cGy ≤ 0.5 cm^3^ was exceeded in the first and third non-adapted plans (1.43 cm^3^ and 1.45 cm^3^, respectively) while the adapted plan did not exceed this constraint (0.21 cm^3^ and 0.2 cm^3^, respectively). Online adaptation also preserved PTV coverage with V95% ≥ 95% for all three treatments being higher than non-adapted plans, and GTV coverage remaining within 2% of non-adapted plans (Table 3).

**Table 3:** Planning goals and treatment data of the three adaptive fractions The summary column lists the mean value and standard deviation of the three adapted treatments. Values exceeding the planning goals are bolded. GTV: gross tumor volume; PTV: planning target volume; PTV_OPT: planning target volume optimized (PTV – (Bronchus Small Airway +0.3 cm) – (Heart/Pericardium + 0.2 cm) – Trachea Large Bronchus); GTV_OPT: gross tumor volume optimized (PTV_OPT – 0.3 cm); Dmax: maximum dose as percent (%) of the prescription dose; Vxx Gy: volume (cm^3^ or %) receiving xx centigray (cGy); D0.04 cm^3^: point volume (0.04 cm^3^) receiving xx centigray (cGy).

Target	Planning Goals	Reference Plan	Scheduled Plan	Adapted Plan	Scheduled Plan	Adapted Plan	Scheduled Plan	Adapted Plan	Adapted Mean	Adapted SD
GTV	V100% ≥95%	90.20%	90.10%	90.20%	90%	89.60%	89.80%	87%	89%	1%
	V95%≥95%	93.30%	93.40%	94.40%	93.30%	94.20%	93.40%	94.10%	94%	0%
	Dmax 0.00 cm^3^ ≤130%	127.70%	127.80%	123%	126.70%	123.40%	124.60%	122.20%	123%	0%
GTV_OPT	V100% ≥98%	99.10%	97.40%	97.60%	97.40%	97.20%	94.30%	93.80%	96%	2%
PTV	D95%≥100%	68.90%	70.60%	69.10%	68.40%	71.90%	65.70%	71.80%	71%	1%
	V95%≥95%	86%	85.30%	85.80%	85.10%	87.10%	82.50%	86.70%	87%	1%
	Dmax 0.00 cm^3^ ≤125%	127.70%	127.80%	123.20%	126.70%	123.40%	128.40%	122.20%	123%	1%
PTV-GTV	D5% <120%	111.60%	111.60%	109.30%	111.80%	109.40%	111.40%	107.10%	109%	1%
Bronchus/small airway	V2580 cGy ≤ 0.5cm^3^	0.46 cm^3^	1.43 cm^3^	0.21 cm^3^	0.81 cm^3^	0.31 cm^3^	1.45 cm^3^	0.2 cm^3^	0.24 cm^3^	0.05 cm^3^
	D0.04 cm^3^ ≤ 3000cGy (1000 cGy per fraction)	970 cGy	1014 cGy	903 cGy	989 cGy	922 cGy	1065 cGy	905 cGy	910 cGy (2730 cGy cumulative)	8.52 cGy
Esophagus	V2790 cGy ≤ 5cm^3^	1.46 cm^3^	1.35 cm^3^	1.30 cm^3^	1.58 cm^3^	0.79 cm^3^	2.31 cm^3^	1.03 cm^3^	1.04 cm^3^	0.21 cm^3^
	D0.04 cm^3^ ≤ 3240cGy (1080 cGy per fraction)	1052 cGy	1032 cGy	1068	1048 cGy	1033	1136 cGy	1024	1042 cGy (3125 cGy cumulative)	18.98 cGy
Great vessels	V3900 cGy ≤ 10 cm^3^	2.54 cm^3^	2.47 cm^3^	1.41 cm^3^	2.35 cm^3^	0.84 cm^3^	2.12 cm^3^	0.59 cm^3^	0.94 cm^3^	0.34 cm^3^
	D0.04 cm^3^ ≤ 4500 cGy (1500 cGy per fraction)	1440 cGy	1420 cGy	1404 cGy	1431 cGy	1389 cGy	1424 cGy	1370 cGy	1388 cGy (4163 cGy cumulative)	13.91 cGy
Heart/pericardium	V2400 cGy ≤ 15 cm^3^	3.19 cm^3^	22.12 cm^3^	4.29 cm^3^	5.61 cm^3^	2.18 cm^3^	5.86 cm^3^	2.35 cm^3^	2.94 cm^3^	0.96 cm^3^
	D0.04 cm^3^ ≤ 3000cGy (1000 cGy per fraction)	978 cGy	1351 cGy	999 cGy	1177 cGy	999 cGy	1195 cGy	999 cGy	999 cGy (2997 cGy cumulative)	0 cGy
Lung	V1140 cGy ≤ 37%	35.90%	36%	36.20%	36.10%	35.30%	35.80%	37.10%	36%	1%
Spinal cord	C1590 cGy ≤ 0.35 cm^3^	0.00 cm^3^	0.00 cm^3^	0.00 cm^3^	0.00 cm^3^	0.00 cm^3^	0.16 cm^3^	0.00 cm^3^	0 cm^3^	0 cm^3^
Trachea/large bronchus	V3900 cGy ≤ 0.00 cm^3^	0.00 cm^3^	0.00 cm^3^	0.00 cm^3^	0.00 cm^3^	0.00 cm^3^	0.00 cm^3^	0.00 cm^3^	0 cm^3^	0 cm^3^
	D0.04 cm^3^ ≤ 4300cGy (1433 cGy per fraction)	1275 cGy	1276 cGy	1265 cGy	1275 cGy	1251 cGy	1274 cGy	1240 cGy	1252 cGy (3756 cGy cumulative)	10.23 cGy
Brachial plexus	V2200 cGy ≤ 3.0 cm^3^	0.00 cm^3^	0.00 cm^3^	0.00 cm^3^	0.00 cm^3^	0.00 cm^3^	0.00 cm^3^	0.00 cm^3^	0 cm^3^	0 cm^3^
	D0.04 cm^3 ^≤ 2600 cGy (866 cGy per fraction)	45 cGy	44 cGy	60 cGy	43 cGy	66 cGy	44 cGy	123 cGy	83 cGy (249 cGy cumulative)	29.39 cGy
Rib	V4000 cGy ≤ 5.00 cm^3^	0.29 cm^3^	0.28 cm^3^	0.03cm^3^	0.38 cm^3^	0.19 cm^3^	0.62 cm^3^	0.01 cm^3^	0.07 cm^3^	0.09 cm^3^
	D0.04 cm^3^ ≤ 5000 cGy (1666 cGy per fraction)	1373 cGy	1374 cGy	1333 cGy	1386 cGy	1357 cGy	1413 cGy	1321 cGy	1337 cGy (4011 cGy cumulative)	14.97 cGy

**Figure 5:**
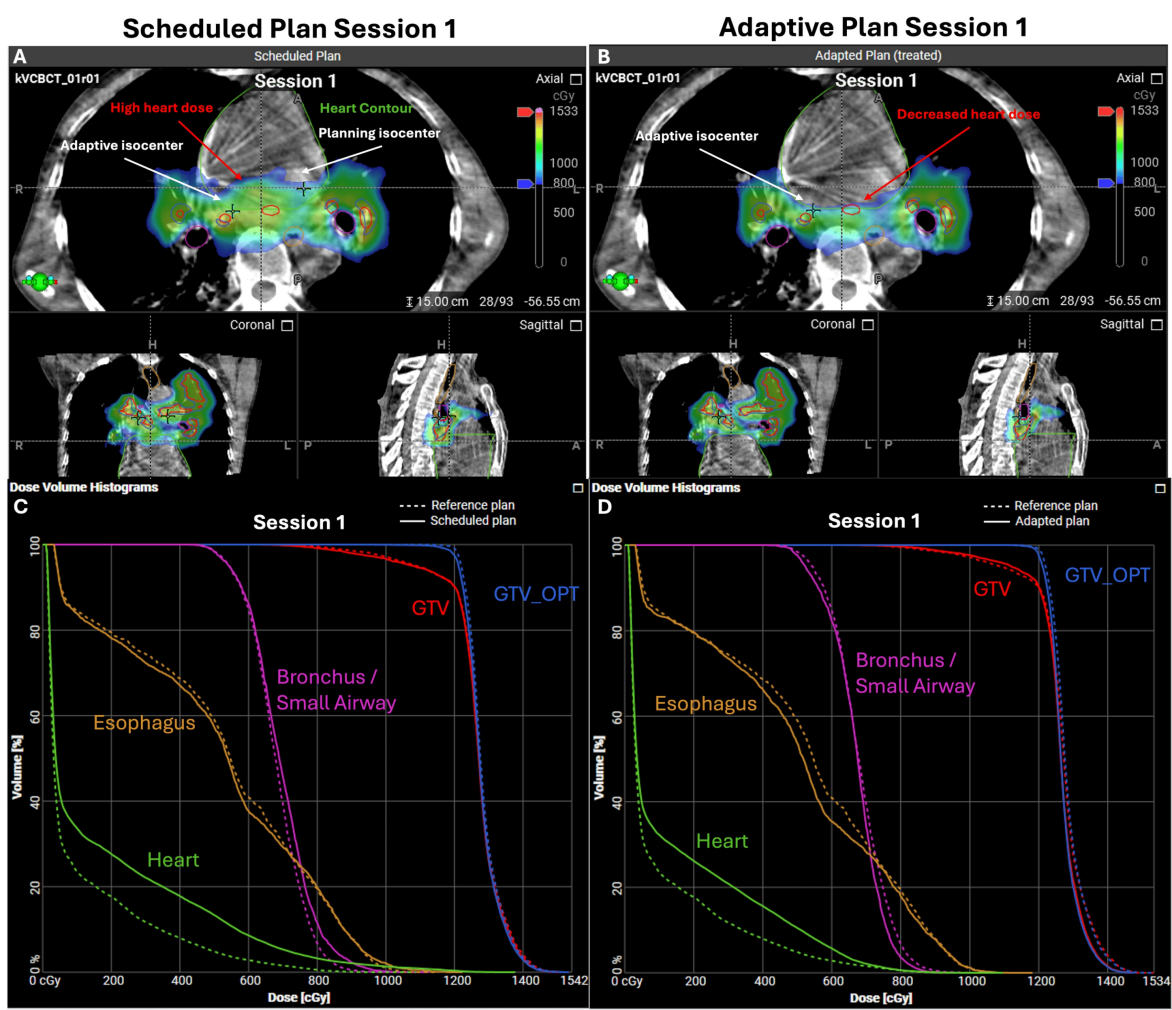
Comparison of reference, scheduled, and adaptive plans on Ethos The cone-beam CT scan and dose color wash of the (A) non-adapted scheduled plan and (B) adapted plan with a cutoff set to 800 cGy per fraction. A comparison of the dose-volume histograms of the dose to the heart (green) in the (C) non-adapted plan shows a greater heart dose (green) in the scheduled plan (solid line) to the reference plan (dashed line). The (D) adapted plan (solid line) showed a lower heart dose compared to the (C) non-adapted plan (solid line) and a smaller increase over the reference plan (dashed line). GTV: gross tumor volume; GTV_OPT: gross tumor volume optimized (PTV_OPT – 0.3 cm); PTV_OPT (planning target volume – (Bronchus/Small Airway +0.3 cm) – (Heart/Pericardium + 0.2 cm) – Trachea Large Bronchus).

The improvements with adaption are reflected visually by comparing high-dose (≥800 cGy) color wash on the cone-beam CT scans and in the dose-volume histograms (DVH) (Figure 5).

## Discussion

This case presents an example of the feasibility of using CT-guided online adaptive treatment to deliver PULSAR [12] with an extended one-month fraction interval as part of consolidative RT for centrally located OM-NSCLC. In fact, one would argue that given the multiple mediastinal nodal regions as well as the bulky lung lesion, the standard SBRT dose and fractionation would have been prohibitive. Tumors are considered centrally located if they are within 2 cm of the proximal bronchial tree, while ultra-central is defined as being within 1 cm [15]. Traditionally, SBRT has been reserved for early-stage NSCLC with a goal biological effective dose (BED) of ≥100 Gy_10_ in one to five fractions, but allowing for up to 10 fractions in large (>5 cm) or central lesions to minimize toxicity [16]. Studies have found high rates of grade 5 toxicity in the treatment of ultra-central tumors with SBRT, which has limited its use in large central lesions [15]. Radiotherapy is increasingly being studied to increase or stimulate an immune response outside of just providing local control [17,18]. SBRT in metastatic/advanced NSCLC to a single tumor site with a dose of 8 Gy in three doses with immunotherapy has been shown to increase the response rate of programmed death-ligand 1 (PDL-1) negative tumors in the PEMBRO-RT trial [19]. It is this proposed remodeling of the tumor microenvironment and release of tumor antigens from radiation to prime the immune system that is an active area of investigation [14,18] and led to the development of the PULSAR timing strategy [12].

Our patient initially presented with a very large span (>16 cm) of his left lung primary and central adenopathy that would not be amenable to traditional SBRT doses. Due to his excellent response to systemic therapy, we found him suitable for consolidative radiation using PULSAR with concurrent immunotherapy to a planned 36 Gy in three one-month interval fractions (BED of 79.2 Gy_10_). An alternative treatment may have been a 15-fraction regimen of a dose of 45-52.5 Gy in 15 fractions. The Ethos^TM^ therapy system allows for direct comparison of the reference plan to the scheduled or an adapted plan based on current anatomy that we found to be most helpful for decreasing the dose to the heart within the acceptable three-fraction SBRT constraints of V2400 cGy ≤ 15 cm^3^ and a point dose of D0.04 cm^3^ ≤ 3000 cGy.

The reference plan developed at the time of CT simulation satisfied all OAR constraints; however, at the time of treatment, the scheduled plan was unable to meet the heart dose constraints. Delivering non-adaptive high dose-per-fraction treatment, in this case, would have most notably increased the risk of acute and late cardiac toxicity for the patient. An adaptive plan was required to meet the heart dose while maximizing the dose delivered to the PTV and GTV. We did note a 17% increase in the size of the heart contour for session one compared to the planned volume that was not seen in treatment sessions two or three, or other target volumes or OARs (Table 2).Upon review, this difference was due to more heart being visible on the CBCT of the first fraction, likely due to cardiac motion. Since the inferior border of the heart was not visible in any of the three sessions of CBCTs, the inferior border was kept consistent based on the reference plan, resulting in a greater heart volume in the first session. Although this had no impact on our cardiac dose constraints, future potential ways to prevent this could be to ensure that the entire heart is visualized in the CBCT or by maintaining a consistent total heart volume in each session.

Additionally, although the esophagus dose constraint of V2790 cGy ≤ 5cm^3^ was met in both the scheduled and adapted plans, the CT-guided online adaptive planning was able to further decrease radiation dose to the esophagus particularly in the second (0.81 cm^3^ vs. 0.31 cm^3^) and third sessions (2.31 cm^3^ vs. 1.03 cm^3^), respectively (Table 3). Lastly, despite two months passing between the first and final RT fractions, we maintained appropriate coverage to GTV, GTV_OPT, and PTV without a need for repeated CT simulation. Both the non-adaptive scheduled plan and adaptive daily plan satisfied our constraints to the great vessels, spinal cord, trachea, rib, skin, and brachial plexus.

Overall, the ability to perform a direct plan comparison on day-of-treatment, and modify it accordingly with adaptive radiotherapy, allowed us to safely deliver a high dose-per-fraction RT to this large centrally located OM-NSCLC.

## Conclusions

This case further demonstrates the feasibility of utilizing adaptive radiotherapy for large centrally located NSCLC and shows that on-treatment adaptation can be used to address inter-fractional anatomical changes even with periods of one month between treatments used in PULSAR. Additional randomized clinical trial data are needed to determine if extended fractionation schedules will improve response to ablative radiotherapy, both with and without the addition of immunotherapy.
